# REGIONAL VARIATIONS IN ENGAGEMENT IN ADVANCE CARE PLANNING AMONG RACIALLY AND ETHNICALLY DIVERSE OLDER ADULTS

**DOI:** 10.1093/geroni/igz038.1017

**Published:** 2019-11-08

**Authors:** Kyeongmo Kim, Michin Hong, Giyeon Kim

**Affiliations:** 1 Virginia Commonwealth University, Richmond, Virginia, United States; 2 Indiana University, Indianapolis, Illinois, United States; 3 Chung-Ang University, Seoul, Korea, Republic of

## Abstract

Advance care planning (ACP) has positive effects on the quality of end-of-life of older adults. Given the influence of environmental factors on health and behavioral health behaviors among older adults, geographic variations may exist in engagement in ACP. This study examined whether 1) there was a regional variation in engagement in ACP and 2) there was a racial/ethnic difference in ACP across regions (Northeast, Midwest, South, and West). Drawn from the 2012 National Health and Aging Trends Study, 2,015 Medicare beneficiaries in the U.S. were included in analyses. Results from logistic regression analysis showed that older adults living in the West (OR=1.66, p=0.003) and the Midwest (OR=1.39, p=0.032) were more likely to be engaged in ACP compared to those living in the South. African Americans (OR=0.31, p<0.001), Hispanics (OR=0.30, p<0.001), and other races (OR=0.49, p=0.04) were less likely than their white counterparts to be engaged in ACP. We also conducted four separate logistic regression analyses by regions. In the Northeast, Midwest, and South, African Americans were less likely to be engaged in ACP compared to whites. In the West, Hispanics were less likely to be engaged in ACP compared to whites. Findings from this study provide a clear picture of racial/ethnic disparities in ACP across different regions in the U.S., suggesting where to target for future interventions to improve engagement in ACP among racial/ethnic minorities. Future research should be conducted at lower levels of geographic areas to find modifiable geographic factors to improve engagement in ACP among older adults.

